# A new step-wise surgical technique of knapsack-like uterine compression sutures for intractable postpartum hemorrhage in cesarean section

**DOI:** 10.1186/s12884-023-06208-x

**Published:** 2024-01-02

**Authors:** Lei Han, Baolin Zhang, Huishu Xu, Hongmei Yin, Yiwei Pang, Xianghui Zhang, Qingliang Zhai, Xiaofeng Liu, Yanlin Wang, Caiying Zhang, Yingjiang Xu, Yanni Liu, Xuemei Chen

**Affiliations:** 1 Department of Reproductive Medicine, Binzhou Medical University Hospital, Binzhou City, 256603 Shandong Province P. R. China; 2 Department of Obstetrics and Gynecology, The Third Affiliated Hospital of Chongqing Medical University, Chongqing, 401120 China; 3 Department of Obstetrics and Gynecology, Binzhou Central Hospital, Binzhou City, 251700 Shandong Province P. R. China; 4 Department of Obstetrics and Gynecology, Binzhou Medical University Hospital, Binzhou City, 256603 Shandong Province P. R. China; 5 Department of Postgraduate Student Office, Binzhou Medical University Hospital, Binzhou City, 256603 Shandong Province P. R. China; 6 Department of Interventional Vascular Surgery, Binzhou Medical University Hospital, Binzhou City, 256603 Shandong Province P. R. China

**Keywords:** Postpartum hemorrhage, Uterine compression suture, Cesarean section, Uterine atony, Placenta previa, Placenta percreta, Placenta accrete

## Abstract

**Background:**

Intractable postpartum hemorrhage (PPH) during cesarean section has been a significant concern for obstetricians. We aimed to explore the effectiveness and safety of a new type of uterine compression suture, the step-wise surgical technique of knapsack-like sutures for treating intractable PPH caused by uterine atony and placenta factors in cesarean section.

**Methods:**

The step-wise surgical technique of knapsack-like sutures was established on the basis of the artful combination of vertical strap-like sutures and an annular suture-ligation technique. This novel surgical technique was applied to 34 patients diagnosed with PPH during cesarean section due to severe uterine atony and placental factors in our department. The hemostatic effects, clinical outcomes and follow-up visit results were all reviewed and analyzed.

**Results:**

This new uterine compression suture successfully stopped bleeding in 33 patients, and the effective rate was 97.06%. Only 1 patient failed and was changed to use bilateral uterine arterial embolization and internal iliac artery embolization. The follow-up visits indicated that 33 patients restored menstruation except for 1 who was diagnosed with amenorrhea. The gynecological ultrasound tests of all the patients suggested good uterine involutions, and they had no obvious complaints such as hypogastralgia.

**Conclusions:**

This step-wise surgical technique of knapsack-like uterine compression sutures can compress the uterus completely. It is a technique that can conserve the uterus and fertility function without special equipment in caesarean section for PPH, with the characteristics of being safe, simple and stable (3 S) with rapid surgery, reliable hemostasis and resident doctor to operation (3R).

## Introduction

Postpartum hemorrhage (PPH) is one of the most common causes of maternal deaths worldwide. The current maternal mortality of PPH is approximately 40 per 100,000 maternities, although it has sharply fallen during the past several decades. It is estimated that PPH can lead to a woman’s death every ten minutes [1, 2]. Uterine atony and placental factors (including placenta previa, percreta and accreta) represent two major causes of PPH, with proportions of approximately 70% and 10%, respectively [1, 3]. They usually occur in and are managed by cesarean Sect [4].

The first-line treatments of PPH include uterine massage and drug therapy, such as oxytocin, misoprostol and carboprost trometamol [5]. However, they exert limited effects in intractable postpartum hemorrhage caused by severe uterine atony and placental factors in cesarean section. As the second-line treatments of PPH, intrauterine tamponade, local suture-ligation, uterine artery ligation, and uterine arterial embolization are often performed if the patients are unresponsive to the first-line treatments [6, 7]. However, these second-line treatments often require proficient surgical techniques of obstetricians, high-end equipment of hospitals, or high medical costs of patients. Hysterectomy is the last resort for the management of PPH because it brings about the loss of fertility and has a large psychological effect on the patients [8].

Uterine compression suture techniques for PPH were developed in the 1990s, including the B-Lynch suture [9], modified B-Lynch sutures [10], Hayman suture [11], and Cho suture [12]. Among these uterine compression suture techniques, the B-Lynch suture is the earliest and most widely used method all over the world [13, 14]. However, in our clinical practice, we find that the applications of the B-Lynch suture or the modified B-Lynch suture alone cannot reach the same ideal effects as previous reports. Therefore, we established a new step-wise surgical technique for intractable PPH caused by both uterine atony and placental factors in cesarean section. This technique is an artful combination of vertical strap-like sutures as well as an annular suture-ligation technique. After suturing, the uterus looks like a knapsack; hence, it is named the knapsack-like uterine compression suture. This new simple technique has been employed to compress the uterus tightly and reduce uterine hemorrhage rapidly and safely in our hospital for intractable PPH patients caused by uterine atony and placental factors during cesarean section. It is easy to master by resident obstetricians and to promote in primary hospitals.

## Subjects and methods

### Subjects

The novel knapsack-like uterine compression suture technique was established in our department in 2016. In this retrospective study, all the cases with performance of cesarean section were reviewed from January 2017 to December 2020 in the Department of Obstetrics and Gynecology, Binzhou Medical University Hospital. The medical cases of the patients who underwent cesarean section because of different gestational complications or specific disorders were reviewed. The diagnostic criteria of PPH were defined as an amount of bleeding ≥ 1000 ml within 24 h after delivery by cesarean Sect [5]. The patients enrolled were either diagnosed as PPH or bled more than 800 ml after first-line therapy and still had the tendency to bleed due to severe uterine atony, placenta previa, placenta percreta or placenta accrete. Their surgical treatments were given special attention. The patients did not have any hematological diseases or coagulation-fibrinolytic system dysfunctions that could affect the hemorrhage in cesarean section. The results of their blood routine test and coagulation function test before the operation were at normal levels.

The maternal, neonatal and follow-up data of these medical cases were collected and analyzed. The maternal data included the causes of operations and PPH, age, parity, gravidity, gestational duration, estimated blood loss, volume of blood transfusions, surgical methods and results of blood tests. The neonatal data mainly referred to the body weights and Apgar scores of the newborns. The surgical design, procedure and informed consent for this study were all approved by the Research Ethics Committee of Binzhou Medical University Hospital.

### Pretreatments before uterine compression suture

The cesarean section was performed using an abdominal Pfannenstiel incision or the previous cesarean section incision under combined spinal epidural anesthesia. When the patients with PPH showed tendencies to bleed excessively, the first and second-line treating protocols were performed immediately after delivery [15, 16]:

1. uterine massage;
2. oxytocin treatment (10 IU fundus uteri injection and 10 IU intravenous drip mixed in 100 ml of 0.9% sodium chloride solution);
3. 10% calcium gluconate, 10 ml slow intravenous injection;
4. carboprost trometamol, 250 µg fundus uteri injection;
5. manual removal of placenta and curettage of uterus;
6. removal of residual placenta and reduction of active bleeding of placenta factors by figure-of-eight suture;
7. repair the uterine incision to the normal morphology of uterus;
8. bilateral uterine artery ligation (if necessary);

### The step-wise surgical technique of knapsack-like uterine compression sutures

There were 34 patients who did not respond to the first-line treatments, so we undertook surgical management using modified uterine compression suturing in a knapsack-like pattern to conserve the uterus with the informed consents of the patients and their families. All the sutures were performed with 1^#^ coated VICRYL plus antibacterial sutures (coated polyglactin 910 suture with triclosan) (Ethicon Inc., U.S.A.).

**Fig. 1:**
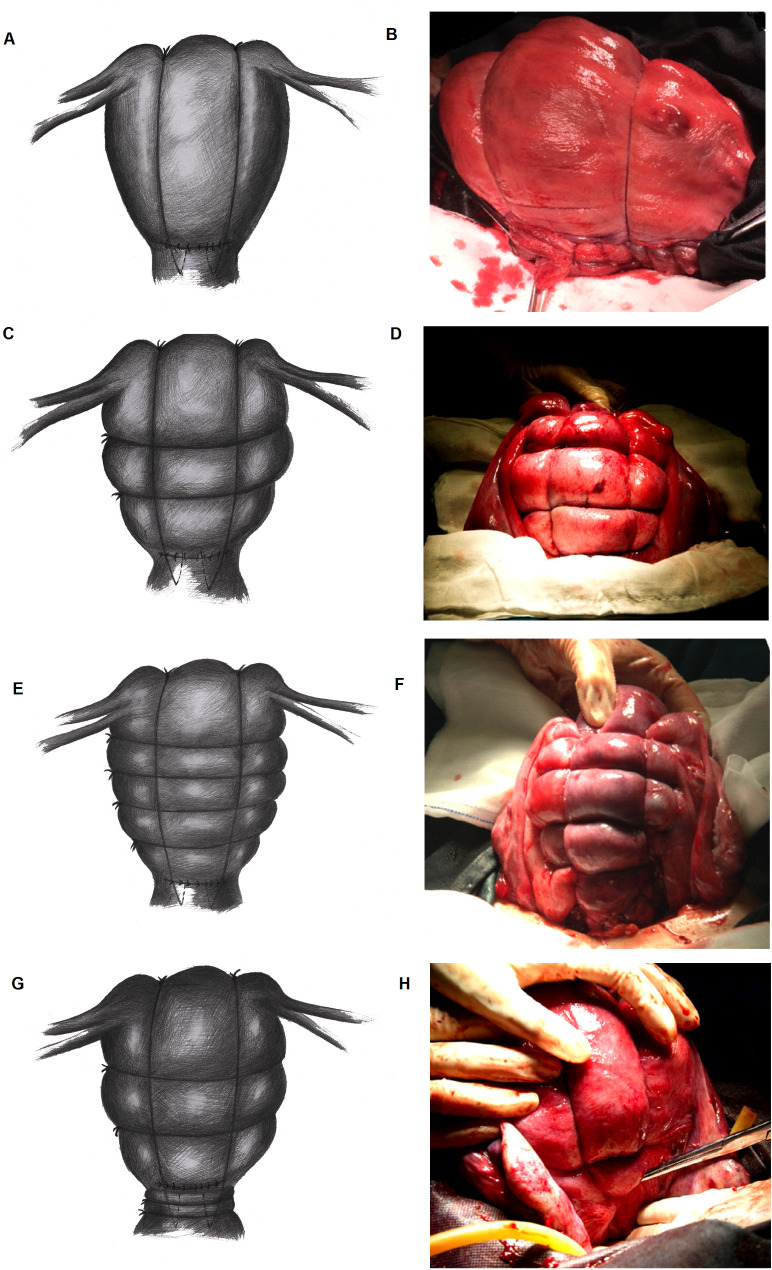
The model and actual pictures of the new step-wise surgical technique of knapsack-like uterine compression sutures

(1) The uterus was exteriorized out of the pelvic cavity and was rechecked carefully to stop bleeding. After confirmation of no obvious bleeding point, the first assistant performed bimanual compressions to the uterus vigorously to stop bleeding and predict the potential hemostatic effects of the compression sutures. If the uterine bleeding was not clearly reduced, the knapsack-like suture procedures were then carried out.

(2) Press down the vesical peritoneal reflection and perform the vertical strap-like suture first (the same as the modified B-Lynch suture [17]): To do this, the first assistant applied forceful bimanual anteroposterior pressure on the corpus uteri continuously. The absorbable suture thread with needle was inserted through the uterus 1 ~ 2 cm below the lower edge of the uterine incision and 2 ~ 3 cm inside the right lateral border of the lower uterine segment. The swaged needle pierced through the uterine cavity from the anterior to the posterior uterine wall and then passed over the fundus uteri. The two ends of the thread were tied at the fundus uteri as tightly as possible. The suture methods of the left lateral border were the same as the above-mentioned approach (Fig. 1A and B).

(3) Carry out the circular suture: The first assistant applied forceful bimanual left-right pressure on the corpus uteri continuously. The swaged needle pierced into the avascular area of parametrium below the left ovarian proper ligament. The thread was circuited around the posterior uterine wall, and the needle pierced out at the axisymmetric position of the entry point. The two ends of the thread were tied in front of the uterine lateral wall tightly and evenly. The strength of the tension should be well-distributed and moderate. Preferably, the loop could accommodate one finger after being tied to avoid insufficient uterine blood supply. If the ligation is too tight, there could be complications of uterine ischemia and necrosis [18, 19]. Thus, the uterus was bundled in a circular pattern for the first circle. After that, the swaged needle pierced into the avascular area of the parametrium below the left uterine round ligament and pierced out at the avascular area below the right ovarian proper ligament. Then, the two ends were tied tightly and evenly to form the second circle of the circular suture (Fig. 1C and D).

(4) Step-wise uterine compression suture: The conditions of uterine bleeding were evaluated again. If the hemostatic effect of the preceding surgical techniques is undesirable, additional sutures are needed above or below the previous circular sutures. The optimal entry point could be chosen at the uterine serosa layer (Fig. 1E and F).

(5) Circular suture of lower uterine segment for patients with placenta factors or lower uterine segment atony: The vesical peritoneal reflection should be pushed down to the level of the external cervical orifice. The entry point of the vertical strap-like suture should be moved down according to the source of the hemorrhage. The needle of the circular suture was then passed through the left frontal uterine serosa layer and came around the posterior wall of the lower uterine segment. Then, the needle was withdrawn from the axisymmetric point of the right uterine serosa layer. The two ends of the thread were tied in front of the lower uterine segment tightly and evenly. The number of circular bundles might increase depending on the hemostatic effect of previous sutures (Fig. 1G and H).

(6) Further hemostasis and observation: After all the sutures and ligations, 1^#^ silk thread was used for sewing hemostasis at the needle hole of the uterus. The conditions of uterine hemorrhage were observed for at least 10 to 15 min to ensure that the amount of vaginal bleeding was significantly reduced and the vital signs of the patients were stable. After that, the abdominal closure was implemented layer by layer.

(1) A and B show the model and actual picture of the vertical sutures at first, respectively. The vertical sutures are the basis of every knapsack-like suture. The absorbable suture thread with needle was inserted through the uterus 1 ~ 2 cm below the lower edge of the uterine incision and 2 ~ 3 cm inside the lateral border of the lower uterine segment. The swaged needle pierced through the uterine cavity from the anterior to the posterior uterine wall and then passed over the fundus uteri. The two ends of the thread were tied at the fundus uteri.

(2) C and D show the model and actual picture of step-wise knapsack-like sutures of another patient who needed 2 vertical and 2 annular circles of sutures in corpus uteri, respectively. The swaged needle pierced into the avascular area of parametrium below the left ovarian proper ligament. The thread was circuited around the posterior uterine wall, and the needle pierced out at the axisymmetric position of the entry point. The two ends were tied in front of the uterine lateral wall. After that, the swaged needle pierced into the avascular area of the parametrium below the left uterine round ligament and pierced out at the avascular area below the right ovarian proper ligament to form the second circle.

(3) E and F show the model and actual picture of knapsack-like sutures of a patient who needed 2 vertical and 4 annular circles of sutures in corpus uteri, respectively.

(4) G and H show the model and actual picture of knapsack-like sutures of a patient who needed 2 vertical, 2 annular circles of sutures in corpus uteri and 2 in the lower uterine segment, respectively. The vesical peritoneal reflection should be pushed down and the entry point of the vertical strap-like suture should be moved down according to the source of the hemorrhage. The needle of the circular suture was then passed through the left frontal uterine serosa layer and came around the posterior wall of the lower uterine segment. Then, the needle was withdrawn from the axisymmetric point of the right uterine serosa layer. The two ends were tied in front of the lower uterine segment. The position and total number of annular (horizontal) circles of step-wise knapsack-like sutures mainly depend on the evaluation of bleeding rate and uterine contraction (hardness of muscular layer) after the implementation of the previous procedure.

### Follow-up procedures

All 34 subjects were informed that they underwent uterine compression suturing in a knapsack-like pattern during their cesarean section and needed to carry out regular follow-up visits at the 42nd day, 3rd month, 6th month, 1st year and 2nd year after delivery. All patients would receive telephone follow-up with the operators every year after delivery. The collected follow-up data included gynecological ultrasound results, duration of lochia, menstrual recovery time, duration of lactation, menstrual blood volume, pregnancy conditions and occurrence of complications.

### Statistical analysis

The variables are shown as the mean ± standard deviation (SD). The statistical significance of parametric variables between two groups was determined using non-paired comparisons performed by *t*-test analysis. Pairwise comparisons were applied to compare the same index of one subject before and after operation, either by Fisher’s LSD for parametric variables or by the Wilcoxon test for non-parametric variables. All tests were 2-sided with 95% confidence intervals (95% CIs). All the data were analyzed using the Statistical Package for Social Sciences version 27.0 (SPSS Inc., U.S.A.). Statistical significances were all determined on the basis of *p* < 0.05.

## Results

### Clinical characteristics of the subjects

**Fig. 2:**
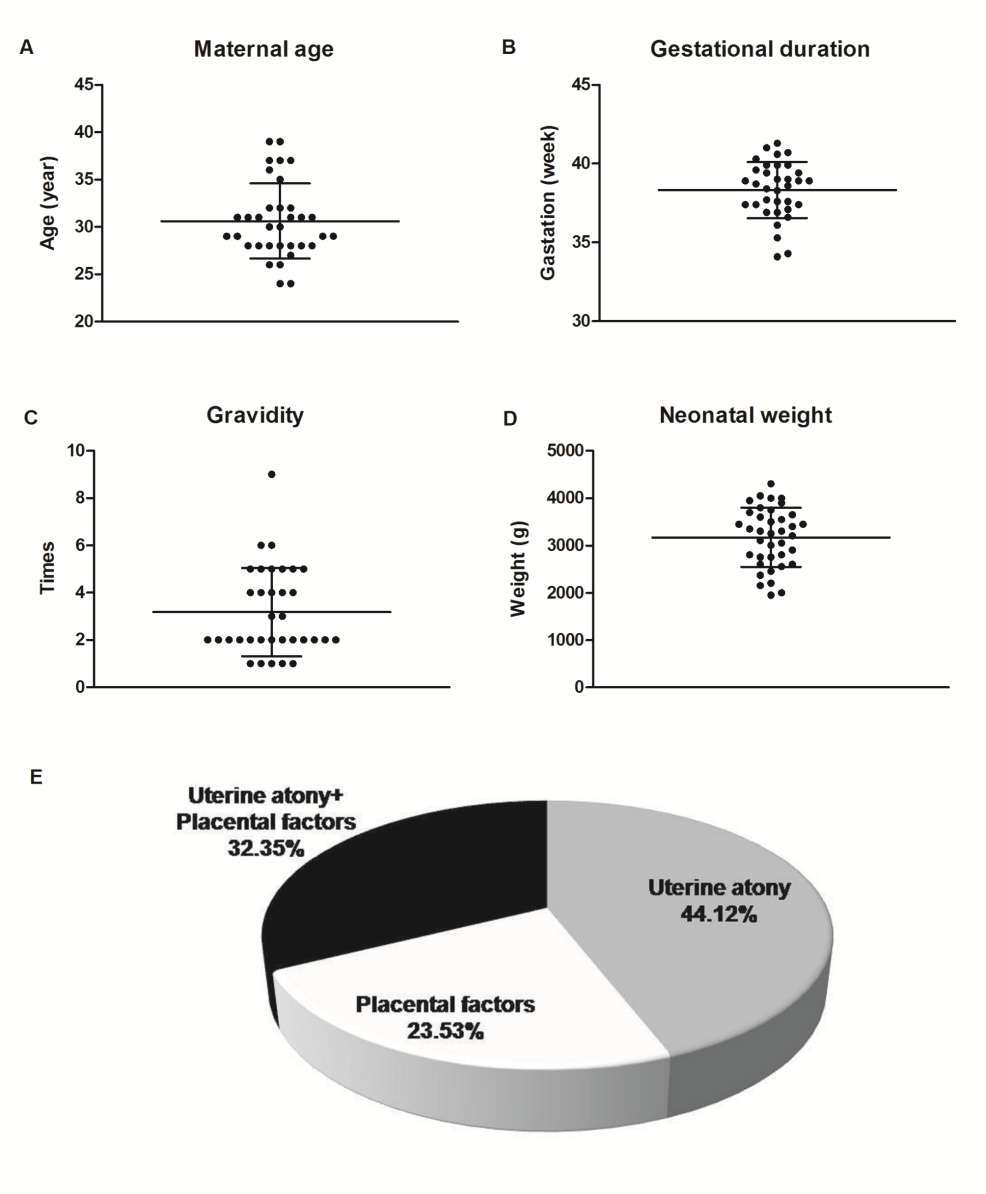
The basic characteristics of the 34 patients. The distributions of maternal ages (**A**), gestational durations (**B**), times of gravidities (**C**), neonatal weights (**D**) and reasons for intractable postpartum hemorrhage (**E**)

All the subjects in this study were reproductive-aged women, and they had an average age of 30.6 ± 4.0 years (Fig. 2A) and gave birth at the gestational durations of 38.3 ± 1.8 weeks (Fig. 2B). Their gravidities ranged from 1 to 9, with a mean value of 3.2 ± 1.9 (Fig. 2C). However, only 7 of these patients were multiparas, and the others were primiparas. There were 4 twin pregnancies among the 34 women. The body weights of the 38 newborns were 3232.0 ± 607.1 g (Fig. 2D), with 17 female and 21 male neonates. Uterine atony was the most frequent indicator for intractable PPH in cesarean section for these pregnant women, accounting for 44.12% of the sample (Fig. 2E). Approximately 32.35% of the patients accepted uterine compression sutures for PPH that resulted from both uterine atony and placental factors (including placenta previa, percreta and accrete) (Fig. 2E). Additionally, 23.53% of the patients who were diagnosed as PPH had the knapsack-like uterine compression suture technique performed because of placental factors only.

### Indications for cesarean section

**Fig. 3:**
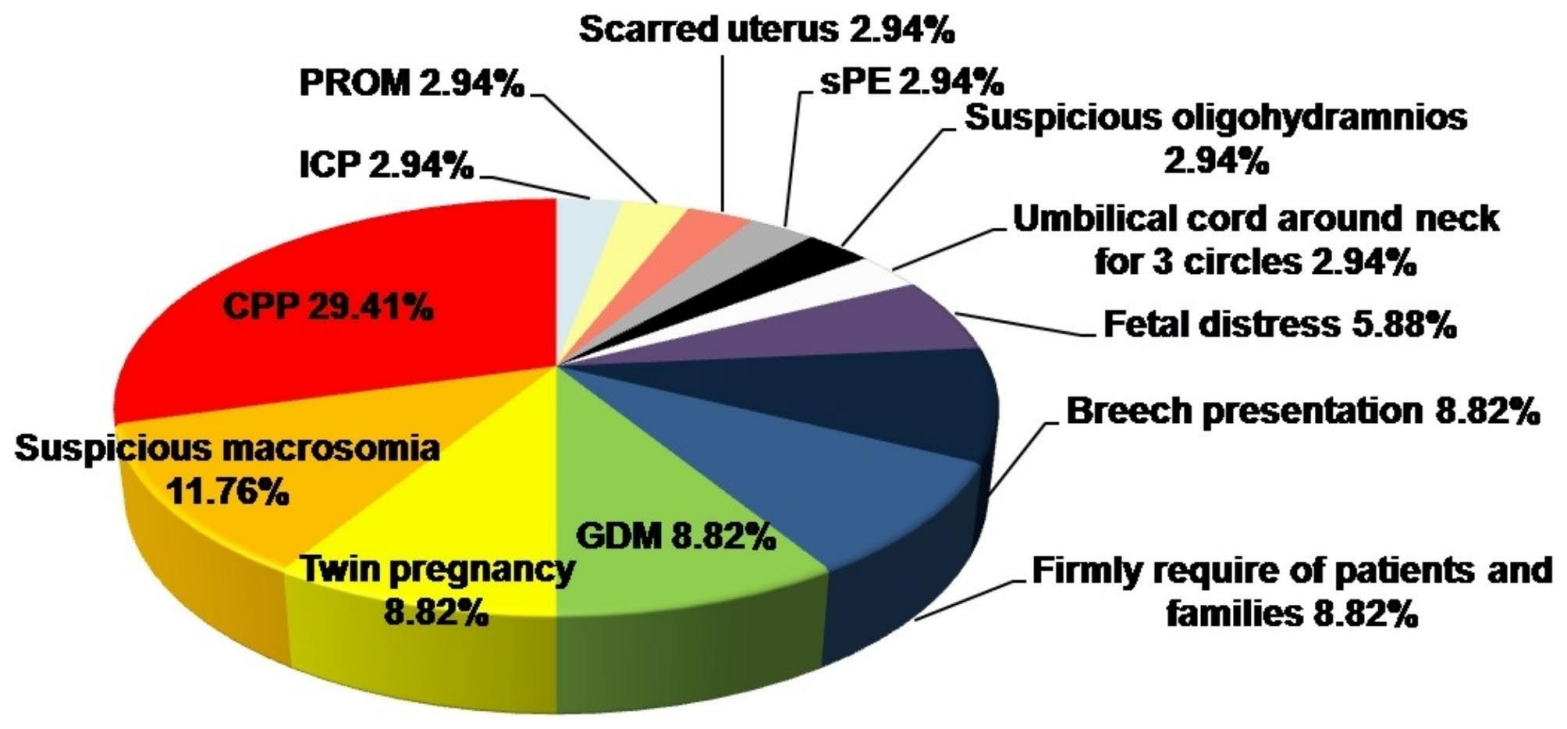
Different operational indications for cesarean sections of the 34 patients. The proportion of each indication was marked beside the gestational complications. Abbreviations: CPP: central placenta previa; GDM: gestational diabetes mellitus; sPE: severe preeclampsia; PROM: premature rupture of membranes; ICP: intrahepatic cholestasis pregnancy

The indications for cesarean section of these patients were various, with different proportions. Among them, the greatest proportion of indications for cesarean section was central placenta previa (CPP), which amounted to 29.41%. Suspicious macrosomia occupied the second-highest proportion, at 11.76%. The proportions of the subjects who underwent cesarean section because of twin pregnancy, gestational diabetes mellitus (GDM), and breech presentation as well as firm requirement of patients and families were each 8.82%. In 5.88% of the patients, cesarean section was performed for fetal distress. The other reasons for accepting cesarean section were 3 loops of the umbilical cord around the fetus’ neck, suspicious oligohydramnios, severe preeclampsia (sPE), scarred uterus, premature rupture of membranes (PROM) and intrahepatic cholestasis pregnancy (ICP), each at 2.94% (Fig. 3).

### Treatments and clinical efficacies

**Fig. 4:**
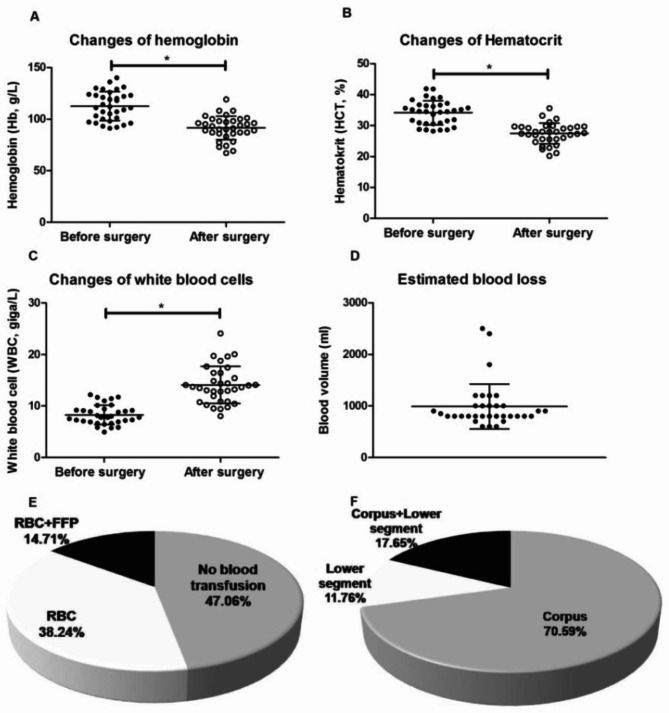
The evaluations of treatment methods and clinical efficacies of the 34 patients. Pairwise comparisons of hemoglobin (**A**), hematocrit (**B**) and white blood cell values (**C**) of all the patients between pre-operation and post-operation (*: *p* < 0.05) are shown in order. The blood losses of these patients were also estimated (**D**). E shows the types (suspended red blood cells and fresh frozen plasma) and proportions of patients with blood transfusions. F shows the locations (corpus uteri and lower uterine segment) and proportions of the patients underwent the new knapsack-like technique of step-wise uterine compression sutures

The routine blood tests of each patient were checked both before and within 24 h after the knapsack-like uterine compression sutures in cesarean sections. The average level of hemoglobin (Hb) of these subjects after the operation was 91.4 ± 11.5 g/L, which was significantly lower than that of subjects before the operation (112.6 ± 14.0 g/L) (Fig. 4A, *p* < 0.05). Similar to Hb, the hematocrit (HCT) of these patients after cesarean section showed a large decrease (27.5 ± 3.4%) compared to before the operation (34.1 ± 3.9%) (Fig. 4B, *p* < 0.05). In contrast, the white blood cell counts after the operation (14.1 ± 3.6 d) were significantly higher than before cesarean Sect. (8.3 ± 1.9 d) (Fig. 4C, *p* < 0.05). The estimated blood loss reached up to 989.7 ± 434.8 ml (Fig. 4D). Nearly half (47.06%) of the patients did not need to have blood transfusions; more than 1/3 (38.24%) of the subjects were given transfusions of suspended red blood cells without leucocytes (Fig. 4E). Only 14.71% of the pregnant women were given the transfusions of both suspended red blood cells and fresh frozen plasma (FFP). Among these patients, approximately 70.59% had the step-wise uterine compression suture performed at the corpus uteri, and 11.76% had it performed at the low segment of the uterus. The step-wise compression sutures were performed at both the corpus uteri and low segment in 17.65% of patients (Fig. 4F).

### Postpartum follow-up visits

**Fig. 5:**
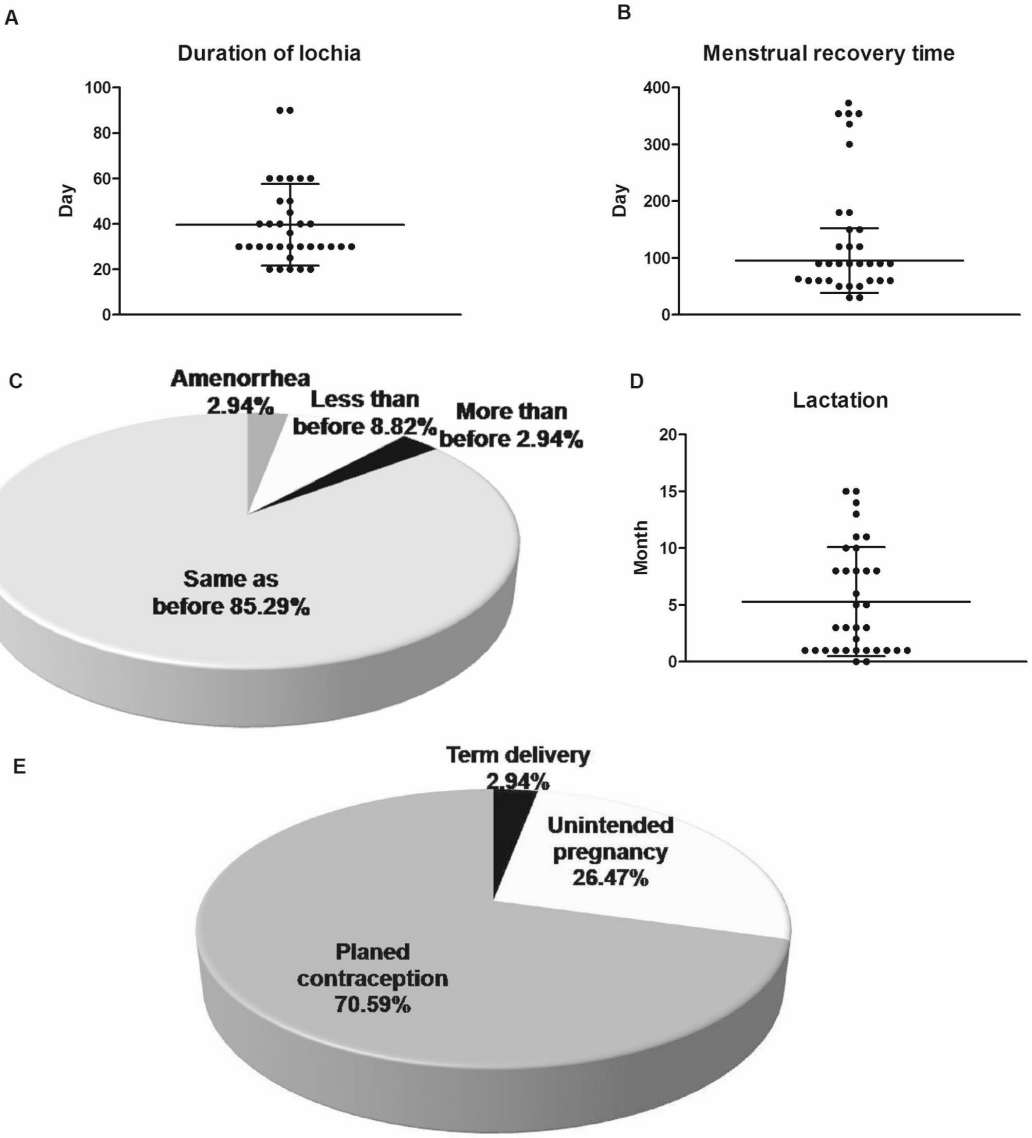
Postpartum recovery conditions of the 34 patients according to the follow-up visits. The distributions of the duration of lochia (**A**), the recovery time of menstrual cycles (**B**), menstrual blood volumes (**C**) and the duration of lactations (**D**) were analyzed. E shows the conditions and proportions of their subsequent pregnancies

Timely postpartum follow-up visits were carried out at different time points after delivery for all the patients. Their gynecological ultrasound tests at the 42nd day postpartum all suggested good uterine involutions. There were no obvious complications reported back from these subjects. Their durations of lochial discharges were 40.0 ± 18.0 days (Fig. 5A). Thirty-three (97.1%) of them took 95.5 ± 57.0 days to restore menstruation (Fig. 5B). To date, only 1 patient (2.9%), who is currently 43 years old, suffered from amenorrhea (Fig. 5C). In February 2023, the serum levels of anti-Mullerian hormone (AMH) of this patient was 1.02 pmol/L (0.14 ng/ml), so we speculate that decreased ovarian reserve may be the main cause of amenorrhea in this patient. The menstrual blood volumes of 29 patients (85.3%) were the same as those before cesarean section (Fig. 5C). There were 3 pregnant women (8.8%) whose menstrual blood volumes were lower during their antenatal periods, and 1 patient (2.9%) found it to be higher than before (Fig. 5C). All of these pregnant women had no obvious hypogastralgia during both their menstrual period and intermenstrual. The lactation durations were 5.3 ± 4.7 months. There were 2 patients who breast-fed their babies for as long as 15 months, but there were also 2 who had given up breast-feeding since delivery because of hypogalactia or crater nipple (Fig. 5D). The patients were all given the medical advice that they should not get pregnant again until 2 years later. Twenty-four patients (70.6%) implemented planned contraception successfully for more than 2 years (Fig. 5E). However, there were still 9 patients (26.5%) who had unintended pregnancies because of contraceptive failures, and they all accepted painless artificial abortions during early pregnancy (Fig. 5E). One of the patients got pregnant and carried to full term and was safely delivered at another hospital with no obvious complications (Fig. 5E).

## Discussion

### Treatment of postpartum hemorrhage

PPH affects approximately 2% of pregnant women and is one of the main reasons for maternal death. According to the data from the WHO, more than 25% of maternal deaths are associated with PPH globally, which is also the leading causes of maternal mortality in developing countries [5]. Uterine atony is the most common pathogenesis of PPH, accounting for 70-80% [20]. The traditional methods that are often used to treat PPH include uterine massage, applications of conservative therapy drugs such as oxytocin, misoprostol, and carboprost trometamol, and local suture ligation, uterine artery ligation, and intrauterine gauze packing [1]. Although uterine artery embolization can be a very important therapy to conserve the uterus, it requires both the conditions of a hospital with emergency interventional operation capability and surgeons with skilled techniques of arterial catheterization and embolization [21]. In addition, stable vital signs and moveable body are necessary preconditions. Therefore, arterial embolism is considered to be especially unsuitable for promotion in primary hospitals. Hysterectomy for saving the maternal life will do a great disservice to the bodies and minds of both pregnant women and their families. It may also lead to a series of medical disputes.

Uterine compression suture techniques, including a variety of new methods, have been in use since the late 1990s for treatment of PPH [22–25]. The B-lynch suture was the first proposed and is the most widely used and the most mature technique [9]. However, in primary clinical cases, we found that the hemostatic effects in some (approximately 20%) patients who had been operated on using the B-lynch or modified B-lynch suture were not as good as the literature reported. The circular uterine suture-ligation techniques were added to control PPH in these patients. The number of circular bundles ranges from 1 to 6, depending on the hemostatic effects in real time. Therefore, we created a new step-wise knapsack-like surgical technique for PPH. This type of uterine compression suture has great advantages in surgical safety, hemostatic effect, operational time and difficulty, and conservation of the uterus and fertility, as well as economic advantages. Moreover, it is easy to master by junior attending obstetricians and to promote in primary hospitals, especially in developing countries, to improve the success rates in treating PPH.

### Hemostatic mechanisms and effects of knapsack-like uterine compression sutures

The main postpartum hemostatic mechanisms depend on strong physiological uterine muscle contraction. The muscle fibers of the myometrium are arranged in different directions and cross arrays. The contraction of uterine muscle fibers will compress and close the blood vessels throughout the myometrium rapidly to achieve hemostasis [26]. On the other hand, massive thrombus formation in the blood sinus of the placenta stripping surface can also stop bleeding [27]. The hemostatic principle of the B-Lynch suture is that mechanical compression in a vertical direction compresses the blood vessels in the uterine wall effectively, reduces and slows down the blood flow significantly, and increases local thrombosis [26, 28]. The hemostatic mechanisms of this new type of knapsack-like uterine compression suture technology are also based on the principle of the B-Lynch suture, and the bilateral longitudinal sutures are applied firstly. Three transversal circular sutures are placed from up (the fundus) to down (the lower segment) of the uterus in Alcides-Pereira sutures [29]. Then longitudinal sutures start posteriorly and end on the anterior aspect of the uterus, with the knot anchored on the lower transverse suture [29]. These methods can also significantly reduce the need for blood transfusion and intensive care unit admission. The mechanical pressures in the vertical and horizontal directions cross each other and effectively compress the blood vessels in the uterine wall and blood sinus of the placenta stripping surface [26, 30]. Some scholars perform compression sutures that do not encircle the entire uterus. Bilateral longitudinal sutures of approximately 5 cm in length are made near the base of the uterus, and transverse sutures are made in the middle and lower uterine segments [31]. The ElNoury-Webster bundle, on the other hand, focuses on low or adherent placentas, and is preceded by a U-shaped full penetration suture of the lower uterine segment after delivery of the placenta, which is another modification of the B-Lynch suture [32]. Removable retropubic uterine compression suture is to fold the body of the uterus into an excessive anterior flexion using absorbable sutures, which are then secured to the abdominal wall behind the pubic symphysis. This may be more effective in stopping hemorrhage in the base of the uterus or in weak contractions of the uterine body, but is highly invasive and there is some controversy over its application [33]. Regardless of the suture method, the local blood flow is rapidly reduced and the bleeding is stopped by the gradual formation of microthrombosis.

This step-wise surgical technique of knapsack-like uterine compression sutures artfully combines vertical sutures and annular suture-ligation techniques, which will press the uterus in all dimensions. Thus, this method is especially suitable for PPH caused by uterine atony because it effectively avoids the treatment failure results from either transversal uterus expansion caused by simple vertical strap-like sutures or longitudinal uterus expansion caused by simple annular sutures [34]. The bilateral uterine artery ligation is no longer needed in our department since we have implemented this new step-wise knapsack-like uterine compression suture technique for intractable PPH in cesarean section. The vertical sutures were carried out to compress the volume of the uterus as soon as possible. The number and position of circular sutures vary with the bleeding rate and hardness of muscular layer at all times. We also extended this novel technique into the treatments of cases of PPH due to placenta accrete or both uterine atony and placental factors (placenta previa, percreta and accreta). The lower segment of the uterus was paid special attention during the suture, because this position is the place where not only placenta previa but also uterine atony is easy to occur. This treatment is safe, simple and stable (3 S) with rapid surgery, reliable hemostasis and resident doctor to operation (3R). Thus it is a good surgical technique to be promoted in primary hospitals, especially in developing countries and areas.

### Key points of the knapsack-like uterine compression suture technique

1. Preoperative assessments of risk factors and sufficient preparations: detailed and thorough physical and accessory examinations should be completed before operation. For those patients with high risk factors of PPH (such as twin pregnancy, polyhydramnios, fetal macrosomia, repeated artificial abortion history, application of tocolytic, thrombocytopenia during pregnancy, pregnancy with hysteromyoma and placenta previa), sufficient preoperative preparations and detailed doctor-patient communications are extremely essential.
2. Accurate measurement of bleeding volume in cesarean section in order to decisively implement the uterine compression suture technique if conventional treatments are invalid for PPH: once the placenta factors complicated by uterine atony are found during cesarean section, immediate uterus massage and conservative drug treatments, rapid suturing of the uterus incision, and local transfixion of the site of placenta accrete or the attachment site of placenta previa should be carried out in sequence. The uterus should be exteriorized out of the pelvic cavity, and the step-wise uterine compression sutures should be performed when the vaginal bleeding continues after the above steps have been implemented. The specific signs include that (1) the uterine contraction does not improve, (2) the uterus appears like a pouch, (3) a large quantity of bright red blood flows out of the vagina when the uterus is squeezed, and (4) the total amount of bleeding has reaches 600 ml. For patients with placenta factors, if the local transfixion of the lower uterine segment is ineffective in hemostasis, the annular suture-ligation can also be performed in the lower uterine segment. There were 10 cases of placenta factors who had this type of suture, and for one of them, the annular suture-ligation for 4 circles was performed. Once PPH occurs, it is necessary to keep in mind that a rubber single lumen drainage tube can be used to bundle the lower uterine segment to block the uterine blood supply, after which the subsequent operations can be performed. This simple approach can effectively avoid a large amount of bleeding from the uterus during surgery.
3. Close observation of vaginal bleeding after suture: it must be observed for at least 10–15 min after suture to ensure that the volume of vaginal bleeding is obviously reduced. The observation includes the volume of blood flowing out of the vagina and vital signs after the extrusion of uterus. The uterus should be squeezed starting from the fundus to the lower segment of the uterus. If there is still a large amount of bright red bleeding or blood clots, the number of circles of annular suture-ligation in the corpus uteri should be increased immediately based on the judgment of the position of bleeding and placental attachment. Cases of placenta factors and lower uterine segment atony should be given the annular suture-ligation in the lower uterine segment before closing the abdomen. Another adequate observation of bleeding is needed after the above procedures. The average total operation time of these 34 cesarean sections was (71.7 ± 21.3) min, which was significantly longer than that of usual cesarean sections in our hospital. This was due to the “step-wised” technique, which requires the operator to carefully use multiple medications or surgeries to stop bleeding and observe their hemostatic effect at each step. There was one failure case in the first year when we established this new technique. Later conclusions showed that a mistake in observing the vaginal bleeding after suturing resulted in the volume of PPH reaching approximately 1000 ml within 2 h after surgery. The patient was treated successfully using bilateral uterine arterial embolization and internal iliac artery embolization.

### The complications of the knapsack-like uterine compression suture technique and its effects on fertility function

It is reported that the complications of uterine compression suture may include infection, late PPH, uterine necrosis, and intrauterine adhesions [6, 14, 20]. However, according to the results of our follow-up visits, there were no obvious complications, such as infection, necrosis and late postpartum hemorrhage, in all 34 subjects. The menstrual cycle of more than 80% of the pregnant women returned to normal within 2 years after deliveries. Only 1 patient, whose is currently 42 years old, was diagnosed with amenorrhea. Although the patients were all given the recommendation to use planned contraception for two years, there were still 9 cases of unintended pregnancies within 2 years after surgery because of contraceptive failures. In addition, there was one patient who got pregnant, carried to full term and delivered again by caesarean section. Therefore, the adverse effects of this method on fertility function were very minimal.

This knapsack-like uterine compression suture technique did not significantly alter menstrual and reproductive outcomes in the majority of patients, which is similar to the effect of previous surgical procedure [23, 35, 36]. Nevertheless, uterine compression sutures carry a higher risk of developing visceral adhesions, recurrent PPH, and the need for repeated compression sutures during subsequent pregnancies [7]. Additionally, patients may be more prone to experiencing distressing memories, negative emotions, and potentially lifelong adverse psychological effects [22]. It was observed that the patients with age < 35 years or received early preventative uterine compression sutures during surgery experienced greater benefit [37].

The combination of intrauterine balloon and uterine compression suture could decrease hemostatic procedures in patients with PPH [38]. It appears to be an ideal fertility and organ-preserving option for patients with intractable PPH, particularly in lower uterine segment bleeding, caused by placenta previa and placenta accreta [39]. If the bleeding is not effectively controlled after pharmacological therapy, balloon compression and various types of compressive uterine suture, the uterine devascularization technique should also be considered [40]. This study does have some limitations, such as the lack of a control group and the fact that the sample was collected in a retrospective manner.

Since the application of the new step-wise surgical technique of knapsack-like uterine compression sutures in our department, the incidence of PPH and the amount of blood transfusion were greatly reduced. If use of the technique spreads, it will also relieve the problem of the lack of blood supply all over the country. Its characteristics can be classified as safe, simple and stable (3 S) with rapid surgery, reliable hemostasis and resident doctor to operation (3R). Supported by our previous clinical results, this new step-wise surgical technique of knapsack-like uterine compression sutures is considered to be an excellent surgical technique and is worthy to be popularized and applied all over the world to conserve the uterus in caesarean sections without special equipment.

## Data Availability

The data that support the findings of this study are preserved in the Administrative Organization of Medical Record of Binzhou Medical University Hospital. But restrictions apply to the availability of these data, which were used under license for the current study and not publicly available. These data can be request from the corresponding authors (Lei Han, Yanni Liu and Xuemei Chen) upon reasonable requirement and with permission of the Administrative Organization of Medical Record and Research Ethics Committee of Binzhou Medical University Hospital.
